# Discussion on Iodism before the French Academy

**Published:** 1860-08

**Authors:** M. Velpeau


### DISCUSSION ON IODISM BEFORE THE FRENCH ACADEMY.

BY M. VELPEAU.

"I have much used," said M. Velpeau, "preparations of iodine, internally and externally. I deem it no exaggeration to affirm that, in twenty-five years, I have administered it to from 12,000 to 15,000 patients. This number will not be considered exorbitant when it is remembered that I exhibit this medicine in the hospital and in my private practice, in the space of a year, to more than 400 persons affected with hydrocele, serous cysts, bursæ mucosæ, sinuses, sanguineous or gangrenous ulcers, etc.; also in refractory tumors, scrofulous adenitis, cancerous affections, caries, tubercular disease of the bones, etc., etc.

"I must premise these remarks by declaring that I have never observed the formidable symptoms which Mr. Rilliet and other practitioners of Geneva have described under the denomination of iodism. I have noticed in a great number of subjects, the phenomena pointed out by Lugol; irritation of the primæ viæ, coryza, obstruction of the nose, salivation; but, I repeat, never those general symptoms bordering on marasmus, never atrophy of certain glandular organs, which appear so common in Geneva. In two cases only, in a young man and women, whom I treated by iodide of potassium, I noticed rapid emaciation, but without any other injury to health. Suspension of the remedy sufficed to restore regularity of nutrition and return of flesh.

"I cannot explain the extraordinary facts pointed out by Mr. Rilliet, but I do not therefore by any means deny them. I have too much confidence in our eminent fellow-practitioner's talents of observation, not to be convinced of the reality of his assertions and of the high degree of attention to which they are entitled.

"I am less well satisfied with his interpretations. I cannot admit that constitutional iodism can be connected either with the absence of iodine in the atmosphere of Geneva; or with the presence of goitre in the patients who came under Mr. Rilliet's observation. Might not more natural explanations be found in the doses of the medicine, in its possible adulteration, and especially in the enormous number of patients subjected to the treatment?

"I will not undertake to solve the question of the influence of doses. That iodine should occasion formidable accidents in infinitesimal doses, and should be, as it were, innocuous in enormous quantities, is a circumstance difficult to explain, in the present state of our knowledge; but it is not a fact that should surprise us, since every day the most usual agents of therapeutics afford us analogous instances. How is it that conjunctivitis is promptly cured by instilling, for four or five days, into the eye one drop of a solution of 1 gr. of nitrate of silver in 1 oz. of water? How is it that calomel, administered in very small doses, according to Law's method, and mercurial frictions on the skin, which allow but infinitely small proportions of mercury to penetrate into the circulation, produce prompt salivation? But a short time since I observed within a few hours, in a young woman, most intense stomatitis with ptyalism, which lasted ten days, in consequence of a very superficial application of the acid nitrate of mercury to an ulcer of the leg as large as a florin-piece. In presence of these facts, which are familiar to all practitioners, may it not fairly be inquired whether it might not be the same with iodine as with hydrargyric preparations?

"Secondly, I opine that the number of patients subjected to the treatment must be taken into consideration, and this Mr. Rilliet has not done. He has observed twenty-three cases of constitutional iodism, but he does not state in what proportion of patients. This is a consideration of paramount importance; for it is obvious that the result will lose much of its value and all its strangeness, if it has occurred but very seldom in the hundreds, or perhaps thousands of cases under Mr. Rilliet's care. I regret, therefore, that, instead of confining himself to the statement of gross numbers, the physicians of Geneva has not supplied us with statistics accurately recording the proportion between the patients subjected to iodic medication, and those who became affected with iodism.

"Upon the whole, the medical practitioners of Paris have never observed constitutional iodism; those of Geneva have sometimes met with it; to what is this difference referable in the mode of action of the same therapeutic agents? The explanations hitherto brought forward appear to me inadequate. It is a fair subject for study and experiment, and one I deem worthy of the attention and solicitude of the Academy."

